# Rush Medical College

**Published:** 1860-09

**Authors:** 


### RUSH MEDICAL COLLEGE.

At a meeting of the students attending the Summer Course of Rush Medical College, session of 1860, a committee of the class presented the following resolutions, which were unanimously adopted:

*Resolved*, That we have been highly benefitted by the instruction we have received during the term now closed, and that we fully appreciate the earnest and efficient efforts, made by each member of the staff, in their respective departments.

*Resolved*, That the clinical instructions we have received at the City and Marine Hospitals, and at the Chicago Eye and Ear Infirmary, have been a source of practical advantage to us, and that we feel under many obligations to the members of the staff who have had charge of these institutions, also to those who, by daily examinations and personal efforts, have so materially aided us in our studies.

*Resolved*, That we tender our sincere thanks to our instructors, for their uniform kindness and courtesy toward us, and that it shall be our constant endeavor to attain a high position in the profession, thus, in some degree, to reflect the beneficial influence which their example has exerted over us.

*Resolved*, That a copy of the above resolutions be sent to the Secretary of the Summer School, and to the *Chicago Medical Journal*, for publication.

Z. P. HANSON, Chairman.
PERCY McALPINE, M. D.,
ROBERT M. LACKEY,
JOSEPH B. SHERMAN,
AUGUST KEEN,
CASPAR MAHLER,
Committee.

CHICAGO, June 23, 1860.

